# Ocular Biometry of Primary Angle-Closure Disease in Younger Patients

**DOI:** 10.3389/fmed.2021.772578

**Published:** 2021-11-03

**Authors:** Shufen Lin, Chengguo Zuo, Yuan Liu, Hui Xiao, Lei Fang, Yihua Su, Liming Chen, Mingkai Lin, Yunlan Ling, Xing Liu

**Affiliations:** ^1^State Key Laboratory of Ophthalmology, Zhongshan Ophthalmic Center, Sun Yat-sen University, Guangzhou, China; ^2^The First Affiliated Hospital, Sun Yat-sen University, Guangzhou, China

**Keywords:** ocular biometric parameter, primary angle-closure disease, younger patients, ultrasound biomicroscopy, anterior segment

## Abstract

**Background:** The purpose of this study was to analyze the ocular biometric parameters of primary angle-closure disease (PACD) in younger patients and compare them with those of elderly patients.

**Methods:** This clinic-based, cross-sectional study included 154 eyes of 154 patients with PACD, consisting of 77 eyes of patients aged 40 years or younger and 77 eyes of patients older than 40. The PACD case definition was compatible with the ISGEO definition. Anterior segment parameters were measured by ultrasound biomicroscopy, axial length (AL) and lens thickness (LT) were measured by A-scan ultrasonography measurements, and the thickness of the retina and choroid were measured by optical coherence tomography. The differences in ocular biometric parameters between different age groups were compared by independent sample *t-*tests or Mann-Whitney *U* tests, and the correlation between the parameters and age was analyzed.

**Results:** Compared to older PACD patients, the lens vault(LV),LV/LT and subfoveal choroidal thickness (SFCT) of younger patients were larger, while the peripheral and mean iris thickness (IT), trabecular-ciliary angle (TCA), ciliary body thickness (CBT), AL and LT were smaller (all *P* < 0.01). There was no significant difference in anterior chamber depth, anterior chamber width, pupil diameter, angle opening distance at 500 μm from the scleral spur, anterior chamber angle and iris convexity between the two groups (all *P* > 0.05). AL, LT, IT, TCA and CBT were positively associated with age (all *P* < 0.001), while LV and SFCT were negatively associated with age (*P* = 0.027 and *P* < 0.001, respectively).

**Conclusions:** Compared with elderly patients, younger PACD patients had more anteriorly positioned lenses, thinner and more anteriorly rotated ciliary bodies, thicker choroids, and shorter axial length. These characteristics might be important anatomical bases for the earlier onset of PACD and the higher risk of malignant glaucoma after filtering surgery.

## Introduction

Primary angle-closure disease (PACD) is a group of diseases that mainly affect Asian populations (1–3). It is commonly a disease of older persons and is rare in younger individuals (4–6). Ritch et al. (5) defined “young” as “40 years old or younger” for the first time. Previous studies have found that younger PACD patients had a higher risk of malignant glaucoma after trabeculectomy (7, 8), suggesting that the ocular biometric structure of younger patients might be different from that of older patients. However, most of the current studies on the ocular biometric parameters of PACD have focused on elderly patients (9–16). The characteristics of the ocular biometric structure of younger PACD patients and their differences with those of older patients remain unknown.

In this study, PACD patients were divided into a ≤ 40-year-old group and a >40-year-old group. The ocular biometric parameters of the anterior chamber, anterior chamber angle, iris, ciliary body, retina, choroid, axial length, and lens thickness were measured by ultrasound biomicroscopy (UBM), optical coherence tomography (OCT), and A-scan ultrasonography measurements. We quantitatively analyzed the ocular biometric parameters of younger PACD patients, and compared them with those of elderly patients.

## Methods

### Study Design and Subjects

This retrospective cross-sectional study included patients who were initially diagnosed with PACD by the same glaucoma specialist (XL) between January 2012 and December 2019 in Zhongshan Ophthalmic Center. PACD patients aged 40 or younger were continuously included, while patients >40 years old were randomly selected during the same period. This investigation adhered to the tenets of the Declaration of Helsinki and was approved by the human research ethics committee of Zhongshan Ophthalmic Center of Sun Yat-sen University in Guangzhou (2021KYPJ003).

PACD was diagnosed based on the criteria of International Society of Geographical and Epidemiological Ophthalmology (ISGEO), including primary angle-closure suspect (PACS), primary angle-closure (PAC), and primary angle-closure glaucoma (PACG) (17). PACS was defined as an eye with iridotrabecular contact (ITC) for at least 180 degrees on indentation gonioscopy in the primary position (without peripheral synechiae), with normal intraocular pressure (IOP) and no optic nerve damage. PAC was defined as an eye with ITC for at least 180 degrees on indentation gonioscopy in the primary position, with peripheral anterior synechiae, with or without elevated IOP, and without glaucomatous optic neuropathy. PACG was defined as PAC together with chronically elevated IOP and evidence of glaucomatous optic changes (defined as a vertical cup-to-disc ratio [CDR] > 0.7 and/or CDR asymmetry > 0.2), and with glaucoma hemifield test outside normal limits (17). The right eye in each subject was selected for analysis if both eyes met the inclusion criteria. Patients with blurred UBM images, secondary factors that can cause angle-closure glaucoma, a history of laser therapy or incisional surgery or eye trauma, a history of acute angle-closure crisis, or a rigid pupil or a change in the pupil shape after long-term use of miotics were excluded.

### Ocular Biometric Measurements

All patients received a comprehensive ocular examination. The best-corrected visual acuity (BCVA) measured with the Snellen chart and converted into a logMAR equivalent. IOP was measured with a Goldmann applanation tonometer. Anterior segments were examined with slit-lamp microscopy, and ocular fundus were examined with a 90 diopter (D) preset lens in a natural pupil state. Anterior chamber angle was analyzed in the dark with Goldmann gonioscopy by the same glaucoma specialist (XL). Axial length (AL) and lens thickness (LT) were measured with A-scan ultrasonography measurements (model US-1800, Nidek), while visual field was measured with Humphrey perimetry (Carl Zeiss Meditec, Dublin). The mean retinal nerve fiber layer thickness (mRNFLT), foveal retinal thickness (FRT), and subfoveal choroidal thickness (SFCT) were measured with optical coherence tomography (OCT; Heidelberg Engineering, Heidelberg, Germany).

### Ultrasound Biomicroscopy (UBM)

The standardized protocol for obtaining UBM images has been reported previously (18, 19). The UBM (model SW-3200L; Tianjin Sower Electronic Technology Co., Ltd., Tianjin, China) examination was performed on the patients by a skilled physician (LC) at the initial consultation. Patients were examined under dark lighting conditions (illumination 60–70 lux) in a supine position. Each eye was assessed with central and 360 degree radiation of the anterior segment. Radial scans were performed at the 12, 3, 6, and 9 o'clock positions centered over the limbus. The images that clearly showed the scleral spur, corneal epithelium/endothelial, iris pigment epithelium, and lens anterior capsule were saved for analysis.

**Figure 1:**
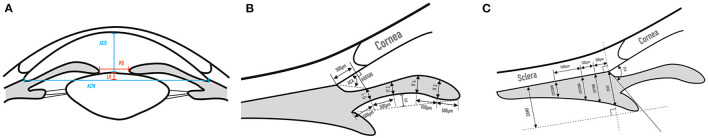
UBM measurement parameters of the anterior segment. **(A)** ACD, ACW, LV, PD; **(B)** AOD500, ACA, IT1, IT2, IT3, IT4, IC; **(C)** TCA, CBT0, CBT500, CBT1000, CBT2000, CBMT.

The UBM parameters of the anterior segment measured in the current study were as follows. Average values of the four quadrants were obtained for all parameters except for anterior chamber depth (ACD) (Figure 1).

1) ACD is the distance from the corneal endothelium to the anterior surface of the lens centered over the pupil (18).
2) The anterior chamber width (ACW) is the horizontal scleral spur-to-spur distance (19).
3) The lens vault (LV) is the perpendicular distance between the anterior surface of lens and the horizontal line joining the two scleral spurs (ACW) (10).
4) The angle opening distance at 500 μm from the scleral spur (AOD500) is the distance between the posterior corneal surface and the anterior iris measured on a line perpendicular to the plain of the trabecular meshwork at 500μm from the scleral spur (18).
5) The anterior chamber angle (ACA) is the angle between the posterior corneal surface and the anterior surface of the iris with the scleral spur as the apex (18).
6) Iris convexity (IC) is the distance from the greatest convexity point on the posterior surface of the iris to the line joining the pupillary margin and the iris root (20).
7) Five parameters of iris thickness (IT) were measured. IT1 is the thickness of the iris at a distance of 500 μm from the root of the iris; IT2 is the thickness of the iris at 1,000 μm from the root of the iris; IT3 is the thickness of the iris at 500 μm from the pupillary margin; IT4 is the thickness of the iris at 1,000 μm from the pupillary margin; and the mean iris thickness (MIT) is the average value of IT1, IT2, IT3, and IT4 (21).
8) The trabecular-ciliary process angle (TCA) is the angle between the most anterior surface of the ciliary body and the posterior corneal surface with the scleral spur as the apex (18).
9) The ciliary body thickness at the point of the scleral spur (CBT0) is the distance between the inner surface of the ciliary body and the inner surface of the scleral at the point of the scleral spur; CBT500, CBT1000, and CBT2000 are ciliary body thickness at 500, 1,000, 2,000 μm from the scleral spur; Ciliary body max thickness (CBMT) is the distance from the most inner point of the ciliary body perpendicular to the inner surface of the sclera or its extended line (18).

All UBM parameters were measured by the same trained physician (SL) who was blinded to the clinical data of the subjects. After an interval of more than 2 weeks, 15 pictures were randomly selected by the same doctor for repeated measurements. The data were evaluated with an intraclass correlation coefficient (ICC) for intraobserver reproducibility (22).

### Statistical Analyses

Statistical analyses were conducted using the statistic package social science version 22.0 (SPSS, Inc., Chicago, IL). The Shapiro-Wilk *W* test was applied for a normality test of the examined parameters. According to the distribution of the data, the differences in parameters between the ≤ 40-year-old group and >40-year-old group were compared using the independent *t*-test or the Mann-Whitney *U* test. The chi-square test was implemented for the qualitative variables. Spearman's rank correlation was used to analyze the correlation between age and ocular biological parameters. The intraclass correlation coefficient (ICC) was applied to evaluate the intraobserver reproducibility. *P* < 0.05 was considered statistically significant.

## Results

### Demographic Data and Basic Characteristics of the Study Subjects

In this study, 1,157 patients with PACD were screened, of which 109 cases were ≤ 40 years old. After excluding patients with a history of acute angle-closure attack (*N* = 15), low-quality UBM images (*N* = 5), and history of miotic use (*N* = 12), 77 eyes of 77 younger patients were included. Seventy-seven eyes of 77 PACD patients older than 40 were randomly selected during the same period. Except for age (*P* < 0.001), there was no significant difference in sex or diagnosis between these two groups (*P* > 0.05) (Table 1). The basic ocular characteristics of various age groups measured by OCT and A-scan ultrasonography measurements are summarized in Table 1. Compared to patients older than 40, younger patients had shorter axial lengths, thinner lenses and thicker SFCTs (all *P* < 0.001).

**Table 1:** Basic clinical data of PACD of different age groups.

**Parameters**	**≤40 years old**	**>40 years old**	***t/Z/*** **χ^2^**	***P***
Eyes	77	77		
Age, years	34.0 (29.0 to 37.5)	58.0 (45.0 to 63.5)	−10.717	**<0.001^‡^**
Male/female	14/63	22/55	2.320	0.128*
PACS/PAC/PACG	9/24/44	4/29/44	2.395	0.302*
BCVA (LogMAR)	0 (0 to 0.3)	0.1 (0 to 0.2)	−0.911	0.362^‡^
IOP (mmHg)	31.06 ± 13.35	29.14 ± 12.26	0.929	0.354^†^
PAS (degrees)	240.0 (153.0 to 330.0)	180.0 (130.0 to 270.0)	−1.501	0.133^‡^
CDR	0.7 (0.3 to 0.9)	0.6 (0.4 to 0.8)	−0.839	0.401^‡^
MD (dB)	−11.13 (−27.49 to −2.42)	−16.24 (−21.47 to −3.28)	−0.732	0.464^‡^
AL (mm)	21.63 ± 0.78	22.31 ± 0.81	−5.231	**<0.001^†^**
LT (mm)	4.68 (4.39 to 4.92)	5.10 (4.84 to 5.37)	−5.622	**<0.001** ^‡^
RNFLT (μm)	73.0 (48.0 to 110.3)	86.0 (53.3 to 106.8)	−0.319	0.749^‡^
FRT (μm)	181.8 (170.6 to 194.0)	188.0 (175.0 to 200.4)	−5.781	0.241^‡^
SFCT (μm)	423.0 (341.0 to 512.0)	291.0 (231.0 to 392.3)	−5.273	**<0.001** ^‡^

*(mean ± SD) or (median, IQR)*.

*IQR = interquartile range, shown as (25%, 75%)*.

† *Independent-Samples t- test*.

‡ *Mann-Whitney U test*.

* *Chi-square test*.

*P-values with statistical significance (< 0.05) are highlighted in bold*.

### Parameters of Anterior Chamber, Iris, and Ciliary Body

**Figure 2:**
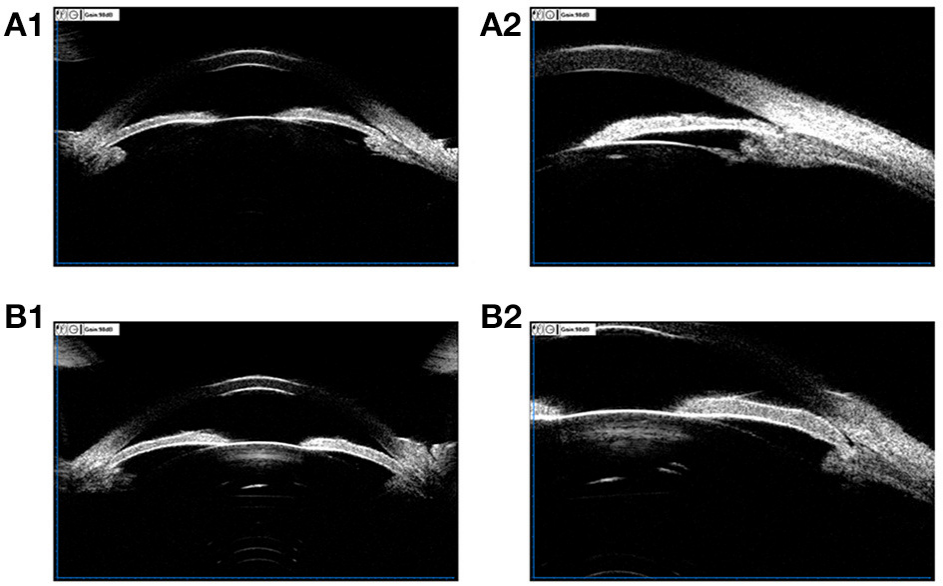
**(A1,A2)** UBM images of a 36-year-old female PACG patient. ACD 1.93 mm, ACW 10.77 mm, LV 0.869 mm, PD 2.17 mm, AOD 0.02 mm, ACA 2.1°, IT1 0.352 mm, IT2 0.338 mm, IT3 0. 459 mm, IT4 0.460 mm, MIT 0.402 mm, IC 0.215 mm, TCA 41.0°, CBT0 0.981 mm, CBT500 0.856 mm, CBT1000 0.710 mm, CBT2000 0.460 mm, CBMT 1.046 mm; **(B1,B2)** UBM images of a 68-year-old female PACG patient. ACD 2.00 mm, ACW 11.47 mm, LV 0.915 mm, PD 2.84 mm, AOD 0.02 mm, ACA 2.5°, IT1 0.392 mm, IT2 0.442 mm, IT3 0. 500 mm, IT4 0.554 mm, MIT 0.472mm, IC 0.293 mm, TCA 62.1°, CBT0 0.906 mm, CBT500 0.911 mm, CBT1000 0.674 mm, CBT2000 0.446 mm, CBMT 1.082 mm.

The comparisons of anterior segment biometric measurements detected by UBM between younger and elderly PACD patients are shown in Table 2. Compared to elderly patients, patients aged 40 or younger had thinner MIT and peripheral iris thickness (IT1, IT2) (*P* < 0.01), relatively anterior positioned lenses (LV, LV/LT were larger) (*P* < 0.01), more anteriorly rotated ciliary bodies (TCA was smaller) (*P* = 0.007), and thinner ciliary bodies (CBT0,CBT500, CBT1000,CBT2000, CBMT) (*P* < 0.01) (Table 2, Figure 2).

**Table 2:** Parameters of anterior segment of different age groups.

**Parameters**	**≤40 years old**	**>40 years old**	***t/z***	***P***
ACD (mm)	1.99 ± 0.36	1.96 ± 0.23	0.688	0.492^†^
ACW (mm)	12.01 ± 0.69	11.90 ± 0.56	0.953	0.343^†^
PD (mm)	3.97 ± 1.14	3.85 ± 0.88	0.725	0.470^†^
LV (mm)	1.21 ± 0.22	1.10 ± 0.22	2.811	**0.006^†^**
AOD500 (mm)	0.016 (0~0.034)	0.020 (0.006~0.040)	−1.955	0.051^‡^
ACA (degree)	1.75(0~3.93)	2.28(0.64~4.59)	−1.955	0.051^‡^
IT1 (mm)	0.37 ± 0.08	0.42 ± 0.08	−4.032	**<0.001^†^**
IT2 (mm)	0.43 ± 0.08	0.47 ± 0.08	−2.977	**0.003^†^**
IT3 (mm)	0.50 ± 0.12	0.51 ± 0.10	−0.683	0.496^†^
IT4 (mm)	0.59(0.49~0.62)	0.58(0.54~0.63)	−0.314	0.754^‡^
MIT (mm)	0.47 ± 0.07	0.50 ± 0.06	−2.663	**0.009^†^**
IC (mm)	0.16(0.11~0.23)	0.18(0.12~0.27)	−1.606	0.108^‡^
TCA (degree)	47.40 ± 13.25	53.03 ± 12.17	−2.735	0.007^†^
CBT0 (mm)	0.82 ± 0.13	0.90 ± 0.12	−3.692	**<0.001^†^**
CBT500 (mm)	0.77(0.71~0.82)	0.83(0.75~0.93)	−2.963	**0.003^‡^**
CBT1000 (mm)	0.59 ± 0.11	0.66 ± 0.12	−3.917	**<0.001^†^**
CBT2000 (mm)	0.30 ± 0.05	0.35 ± 0.06	−4.626	**<0.001^†^**
CBMT (mm)	0.96 ± 0.11	1.00 ± 0.11	−2.630	**0.009^†^**
LV/LT	0.26 ± 0.05	0.21 ± 0.04	5.822	**<0.001^†^**

*(mean ± SD) or (median, IQR)*.

*IQR = interquartile range, shown as (25%, 75%)*.

† *Independent-Samples t-test*.

‡ *Mann-Whitney U test*.

*P-values with statistical significance (< 0.05) are highlighted in bold*.

### Correlation Analysis Between Ocular Biometric Parameters and Age

**Figure 3:**
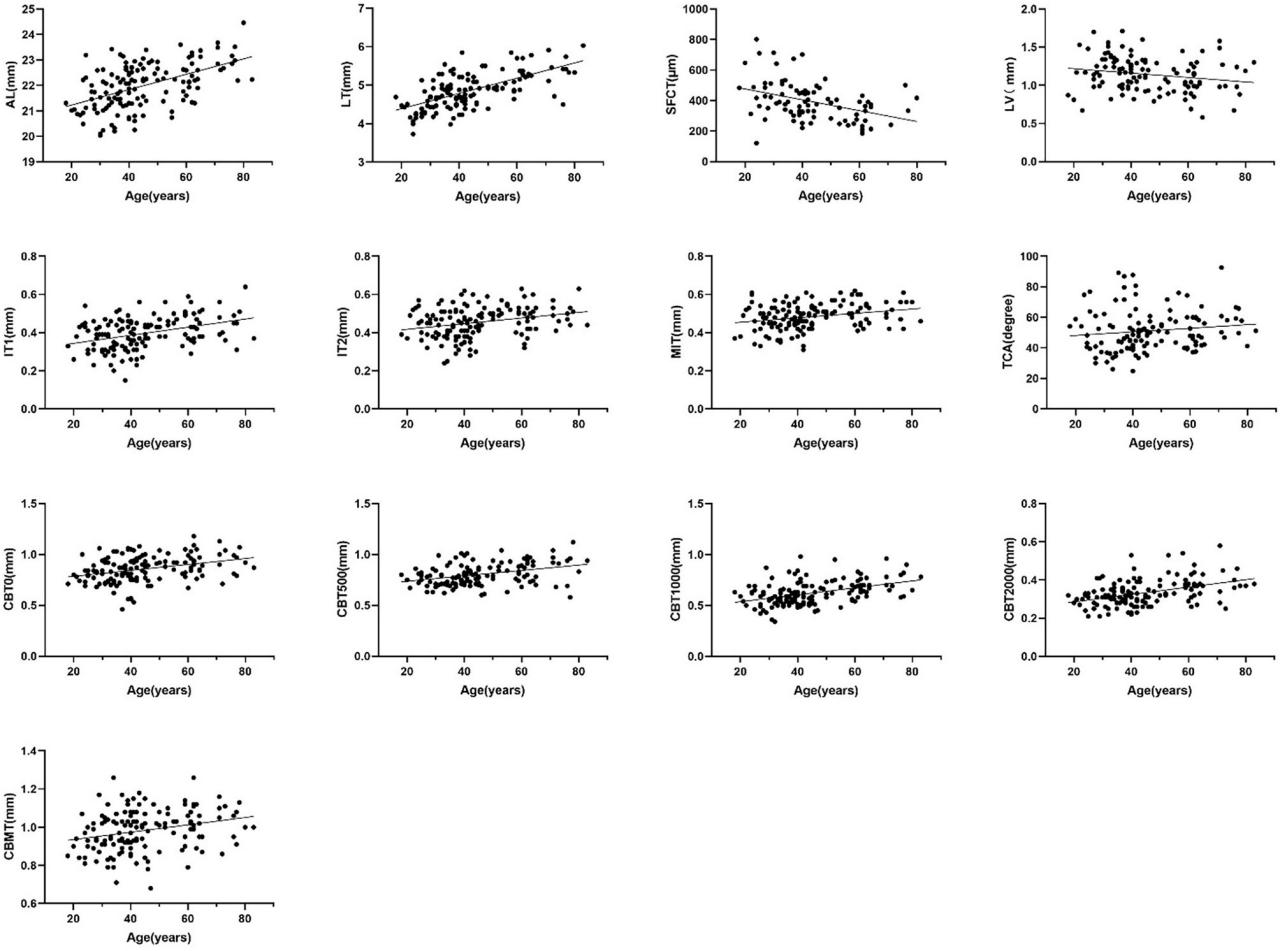
Scatter diagram of the correlation between age and AL, LT, SFCT, LV, IT1, IT2, MIT, TCA, CBT00, CBT500, CBT1000, and CBMT (Spearman's rank correlation, *P* < 0.05).

The current study found that ocular biological parameters including AL, LT, SFCT, LV, IT1, IT2, MIT, TCA, CBT0, CBT500, CBT1000, CBT2000, and CBMT were significantly different between different age groups. The correlation between these parameters and age was analyzed. The result indicated that AL, LT, IT1, IT2, MIT, TCA, CBT0, CBT500, CBT1000, CBT2000, and CBMT were positively associated with age (*r* = 0.501, 0.630, 0.352, 0.268, 0.244, 0.272, 0.357, 0.340, 0.427, 0.391, 0.290, all *P* < 0.001), while LV and SFCT were negatively associated with age (*r* = −0.208, *P* = 0.027; *r* = −0.636, *P* < 0.001) (Figure 3).

### Reproducibility Analysis of UBM Parameters

The UBM images of 15 eyes were randomly selected to analyze the repeatability of the UBM parameter measurement (Table 3), except that the ICC value of CBT2000 was 0.526. All the other parameters verified excellent reproducibility with ICCs (ICC 0.778–0.996).

**Table 3:** Intraclass correlation coefficient (ICC).

**Parameters**	**ICC value**	***P***
ACD (mm)	0.994	<0.001
ACW (mm)	0.9	<0.001
PD (mm)	0.911	<0.001
LV (mm)	0.996	<0.001
AOD500 (mm)	0.954	<0.001
ACA (degree)	0.956	<0.001
IT1 (mm)	0.836	<0.001
IT2 (mm)	0.794	<0.001
IT3 (mm)	0.925	<0.001
IT4 (mm)	0.932	<0.001
IC (mm)	0.904	<0.001
TCA (degree)	0.887	<0.001
CBT0 (mm)	0.918	<0.001
CBT500 (mm)	0.778	<0.001
CBT1000 (mm)	0.888	<0.001
CBT2000 (mm)	0.526	0.02
CBMT (mm)	0.916	<0.001

## Discussion

This study measured the ocular biometric parameters of PACD in younger patients, and compared them with those of older patients. We found that compared with older patients, younger patients had more anteriorly positioned lenses, thinner and more anteriorly rotated ciliary bodies, thicker choroids, and shorter axial lengths. The anatomical characteristics of younger patients might play an important role in the early onset of PACD.

There were obvious differences in anterior anatomical structure between PACD patients and normal people, including short axial length, shallow anterior chamber, narrow angle, and thicker and relatively anteriorly positioned lenses (10–13). However, previous studies that investigated the biological characteristics of PACD mainly focused on older patients. Few systematic study has reported the ocular biometric parameters of young PACD patients or their differences with those of elderly patients.

In this study, we found that the LT of young patients was thicker than that of normal populations of the same age group (3.95 ± 0.30 mm) (23), and the LV was larger than that of normal people (0.419 ± 0.236 mm) (11). This result is in accordance with the findings of previous studies on older patients, which suggested that increased LT and LV were risk factors for angle closure (10, 11, 24). Our study indicated that larger LT and LV also played a part in the onset of PACD in younger patients.

It was interesting to note that while younger patients had shorter ALs and thinner lenses than older patients, their LV and LV/LT were larger, which suggests that the lenses of younger PACD patients significantly moved forward. This result was consistent with the studies of Ozaki et al. (10) and Nongpiur et al. (11), who found a poor correlation between LV and LT. Thus, according to the findings of Nongpiur et al. (10), the increase in LV in younger PACD patients can be related to zonular laxity. Since younger PACD patients were free of abnormal lens zonules due to trauma or other eye lesions, and the zonules of youth were less likely to degenerate (25), the strain of the lens zonules was probably related to ciliary bodies. Gohdo et al. (9) speculated that a thinner ciliary body may cause advancement and thickening of the lens. He et al. (16) believed that the reduced thickness of the ciliary body might be due to muscular atrophy, and a thinner ciliary body might cause anterior positioning of the ciliary processes and loosening of the zonules, resulting in anterior advancement of the lens. Our study found that younger patients had thinner and more anteriorly rotated ciliary bodies than older patients, suggesting that younger patients have inadequate ciliary muscle strength, which is probably the anatomical basis for anterior advancement of the lens in younger patients.

Our study also found that compared with older patients, younger patients had thinner irises and thicker choroids, which is consistent with previous studies in normal populations showing iris thickening with age, while the SFCT thinning with age (12, 26, 27). This result was also in accordance with the findings that choroid thickness and iris thickness were negatively correlated in the studies of Huang et al. (28) and Li et al. (29). Iris, ciliary body, and choroid all originate from uvea that are filled with blood vessels from the same arterial system (ophthalmic artery), so we speculated that blood flow might play an important role in this connection. Both the long posterior ciliary artery (LPCA) and the short posterior ciliary artery (SPCA) stem from the ophthalmic artery. The thinner iris and ciliary body might represent smaller vascular diameters and increased blood flow resistance of the LPCA, resulting in increased blood flow of the SPCA, which was characterized by thickening of the choroid (28).

The correlation between ocular biometric parameters and the age of patients with PACD was analyzed in our study. We found that the AL, LT, IT, TCA, and CBT were positively associated with age, while the LV and SFCT were negatively correlated with age. There has been no systematic study that analyzed the relationship between ocular biometric parameters and age in PACD patients, but previous studies of normal populations have shown that the AL,TCA,CBT, and SFCT were negatively correlated with age (12, 16, 26, 27, 30–33), while the LT, LV, and IT were positively associated with age (12, 23). The correlations between age and LT, IT, and SFCT in this study were consistent with those in normal people, but the correlations between age and AL, LV, CBT, and TCA were opposite to those in the normal population. We speculate that the main reason for this inconsistency might be the different study subjects. Previous studies have analyzed the trend of ocular biometric parameters changing with age in normal populations, but in our study, the subjects were two groups of PACD patients onset at different ages, which meant that the ocular structure of older patients was not simply the state of young patients becoming older. These anatomical features of shorter AL, larger LV, and thinner and more anteriorly rotated ciliary body in younger PACD patients which are distinguished from the changing trend in normal people, might together lead to earlier onset of the disease. In addition, according to previous studies (7, 8, 21, 34), these anatomical characteristics in young patients were also risk factors for the occurrence of malignant glaucoma after filtering surgery.

A main limitation of our study was selection bias at baseline. The study was clinic-based and the sample size was not large, and thus may not be representative of the population as a whole. Besides, the different groups of angle closure (PACS, PAC, and PACG) gave a heterogenous group. So further large-scale cohort study and subgroup analyses are needed. It would also be interesting to qualitatively classify the patients into different mechanisms of angle closure and compare their frequency in different age groups.

## Conclusion

In conclusion, the ocular biometric parameters in young patients were significantly different from those of older patients. The characteristics of anteriorly located lenses, thinner and anteriorly positioned ciliary bodies, thicker choroids, and shorter axial lengths in younger patients were probably important anatomical bases for the early onset of PACD and high risk of malignant glaucoma after filtering surgery.

## Data Availability Statement

The raw data supporting the conclusions of this article will be made available by the authors, without undue reservation.

## Ethics Statement

The studies involving human participants were reviewed and approved by the Human Research Ethics Committee of Zhongshan Ophthalmic Center of Sun Yat-sen University in Guangzhou (Ethical No: 2021KYPJ003). Written informed consent for participation was not required for this study in accordance with the national legislation and the institutional requirements.

## Author Contributions

Design and conduct of the study by SL and XL. Collection of the data by SL, LF, ML, YLin, and YS. Analysis of the data by SL, HX, and LC. Preparation of the manuscript SL, CZ, YLiu, and XL. Review and final approval of the manuscript by all the authors.

## Funding

This study was supported by the National Natural Science Foundation of China (81970808), Sun Yat-Sen University Clinical Research 5010 Program (2014016), and Fundamental Research Funds of the State Key Laboratory of Ophthalmology.

## Conflict of Interest

The authors declare that the research was conducted in the absence of any commercial or financial relationships that could be construed as a potential conflict of interest.

## Publisher's Note

All claims expressed in this article are solely those of the authors and do not necessarily represent those of their affiliated organizations, or those of the publisher, the editors and the reviewers. Any product that may be evaluated in this article, or claim that may be made by its manufacturer, is not guaranteed or endorsed by the publisher.
